# Evaluation of Syncope Reveals Cardiac Amyloidosis

**Published:** 2018-08-30

**Authors:** Matthew Lippmann, Rami Zayed, Jennifer Fink

**Affiliations:** 1 Department of Internal Medicine, University of Kansas Medical Center, Kansas City, KS; 2 Division of Cardiovascular Diseases, University of Kansas Medical Center, Kansas City, KS

**Keywords:** amyloidosis, hereditary, transthyretin-related, syncope, plasma cell dyscrasia

## INTRODUCTION

Tissue deposition of protein fibrils causes a group of rare diseases called systemic amyloidosis.^1^ The most frequent type in high-income countries is AL amyloidosis, which is an acquired systemic immunoglobulin light chain amyloidosis. There is a paucity of epidemiological data for this systemic disease. The first population-based study of AL amyloidosis in the United States came out of Olmsted County, MN, and was published in 1992.^2^ It reported the incidence of AL amyloidosis as three to five cases per million population.

This case report and literature review is intended to increase clinicians’ awareness about this disease because early diagnosis of AL amyloidosis will have significant impact on a patient’s morbidity and mortality. We describe a patient who presented with the primary concern of syncope secondary to cardiac amyloidosis (AL-type).

## CASE REPORT

An 81-year-old male with a past medical history of congestive heart failure with preserved ejection fraction, paroxysmal atrial fibrillation, monoclonal gammopathy of undetermined significance (MGUS), carpel tunnel syndrome, peripheral neuropathy, and chronic transudative pleural effusions was referred to the hospital by his primary care physician with a chief complaint of syncope, which had been getting progressively worse over the prior two months. He also complained of weakness, fatigue, chronic diarrhea, numbness, tingling, easy bruising, and easy bleeding. The patient listed only two medications that he was taking, warfarin and vitamin D supplementation.

On admission, he was afebrile. His blood pressure was 160/80 mmHg, heart rate 91 beats per minute, respirations of 22 breaths per minute, and he was 99% SpO2 on room air. Orthostatic vitals were taken: sitting blood pressure 147/86 mmHg and pulse 89 bpm; standing blood pressure was 118/66 mmHg and pulse 100 bpm. He was awake, alert, and oriented. No scleral icterus was present. He had jugular venous distention. Grade II/VI systolic ejection murmur was noted at the right upper sternal border and 2nd intercostal space without radiation to the carotids. Diffuse crackles were present but no wheezes. There was no labored breathing.

On abdominal examination, there was no tenderness to palpation, normoactive bowel sounds were present, and no masses or hepato-splenomegaly were palpated. Rectal exam revealed a weak rectal tone without masses, fissures, or hemorrhoids. There was 1+ pitting edema to the mid-anterior shin bilaterally without cyanosis or clubbing. There were multiple purpuric, non-pruritic, non-blanching purplish lesions noted on the right neck and lower extremities. Neurological examination revealed no gross cranial nerve abnormalities, strength testing of 5/5 throughout, and normal sensation.

Pertinent lab data revealed normocytic anemia, INR 2.2, creatinine 1.52 mg/dL (no known baseline), urea 32 mg/dL, normal liver function tests, and normal electrolytes. Urine studies showed 2+ protein, 1+ blood, negative nitrite, negative leukocyte esterase, and 2–5 hyaline casts. Brain natriuretic peptide was 1478 pg/mL and troponin mildly elevated at 0.08 ng/mL. TSH, aldolase, cortisol level, erythrocyte sedimentation rate, rheumatoid factor, anti-cyclic citrullinated peptide were within normal limits. Vitamin B12 was low at 276 pg/mL. He had an ANA elevated at 1:640 titer with a speckled pattern.

Chest x-ray revealed an enlarged cardiac silhouette, reticular linear opacities noted bilaterally, but no pneumothorax or alveolar consolidation. CT of the chest revealed reticular sub-pleural fibrosis, no significant honeycombing, and bilateral pleural effusions. Electrocardiogram (ECG) revealed a sinus rhythm, rate in the 90s, and left bundle branch block (baseline unknown); normal voltage criteria were met. A 2-D+Doppler echocardiogram revealed a left ventricular ejection fraction of 45%, Grade III (severe) diastolic dysfunction, moderate concentric bi-ventricular hypertrophy, dilated left and right atria, and no pericardial effusion. He had an echocardiogram four months prior to his admission demonstrating a left ventricular ejection fraction of 60%, moderate concentric left ventricular hypertrophy, and Grade II (moderate) left ventricular diastolic dysfunction). Of note, the patient did not have valvular abnormalities noted on the echocardiogram but auscultation revealed a Grade II/VI systolic ejection murmur. One of the most common ascultatory findings is a systolic murmur and, among elderly patients, the prevalence of systolic murmurs ranges from 29% to 60%, and results of echocardiography are normal in 44% to 100% of cases.^3^ Cardiology was consulted because of concern for cardiac amyloidosis.

Cardiac catheterization revealed that the patient was unlikely to have a restrictive cardiomyopathy, as the diastolic pressures in both the right and left ventricle were below normal. Fat pad biopsy did not reveal amyloidosis. He followed up with outpatient cardiology and underwent implantation of a permanent pacemaker for symptomatic tachycardia-bradycardia syndrome. He also underwent an endomyocardial biopsy that revealed “early stages” of AL amyloidosis. A bone marrow biopsy revealed 8% monoclonal lambda plasma cells, and free light chains showed markedly increased lambda light chains and mildly elevated kappa light chains. Serum protein electrophoresis showed a monoclonal spike in beta/gamma regions. Urine immunofixation revealed IgG lambda. The patient was referred to outpatient oncology where he was started on melphalan and dexamethasone for AL amyloidosis.

## DISCUSSION

Our patient had AL amyloidosis, which is a plasma cell dyscrasia that is related to multiple myeloma^4^ and MGUS.^5^ The major organs commonly involved in AL amyloidosis are the kidney, heart, nervous system, skin, and gastrointestinal system. Our patient likely developed chronic kidney disease secondary to his underlying amyloidosis. Proteinuria, cardiomyopathy, abnormal cardiac conduction, autonomic neuropathy, chronic diarrhea, and capillary fragility due to amyloid infiltration were present in our patient. The severity and number of organs involved determine the prognosis of AL amyloidosis. In fact, cardiac involvement carries the worst prognosis, with a median survival in the untreated patient of about six months from the onset of congestive heart failure.^6^ Death in cardiac AL amyloidosis occurs either as a result of progressive heart failure or sudden cardiac death.^7^

The role of a prophylactic implantable cardioverter-defibrillator (ICD) remains indefinable in preventing sudden cardiac death as pulseless electrical activity is the most common cause of death which is a non-shockable rhythm. Electrocardiogram findings in amyloidosis may lead incorrectly to suspicion of coronary artery disease.^8^ On echocardiogram, wall thickening in amyloidosis is due to infiltration and, unlike true left ventricular hypertrophy in which ECG voltage is increased, the voltage in amyloidosis is low, providing a strong clue to the presence of an infiltrative myocardial disorder.^9^ Understanding what to expect on ECG or echocardiogram in a patient presenting with a constellation of symptoms is important in diagnosing AL amyloidosis.

## CONCLUSION

Prompt recognition of cardiac amyloidosis is important in evaluation of patients with syncope because of its poor prognosis if left untreated. Despite diagnostic modalities such as echocardiogram, computed tomography, and cytogenetic testing, history and physical examination remain the most valuable tools in a physician’s armamentarium. In conclusion, AL amyloidosis must be suspected in patients presenting with syncope in the setting of autonomic neuropathy, proteinuria, cardiomyopathy, chronic diarrhea, and purpura.
